# Multi-Frequency GPR Image Fusion Based on Convolutional Sparse Representation to Enhance Road Detection

**DOI:** 10.3390/jimaging12010052

**Published:** 2026-01-22

**Authors:** Liang Fang, Feng Yang, Yuanjing Fang, Junli Nie

**Affiliations:** 1 School of Artificial Intelligence, China University of Mining and Technology (Beijing), Beijing 100083, China; yangf@cumtb.edu.cn (F.Y.); fyj13141314@163.com (Y.F.); 2 Guizhou University, Guiyang 550025, China; junlinienie@163.com

**Keywords:** road non-destructive testing, convolutional sparse representation, data fusion, multi-frequency GPR

## Abstract

Single-frequency ground penetrating radar (GPR) systems are fundamentally constrained by a trade-off between penetration depth and resolution, alongside issues like narrow bandwidth and ringing interference. To break this limitation, we have developed a multi-frequency data fusion technique grounded in convolutional sparse representation (CSR). The proposed methodology involves spatially registering multi-frequency GPR signals and fusing them via a CSR framework, where the convolutional dictionaries are derived from simulated high-definition GPR data. Extensive evaluation using information entropy, average gradient, mutual information, and visual information fidelity demonstrates the superiority of our method over traditional fusion approaches (e.g., weighted average, PCA, 2D wavelets). Tests on simulated and real data confirm that our CSR-based fusion successfully synergizes the deep penetration of low frequencies with the fine resolution of high frequencies, leading to substantial gains in GPR image clarity and interpretability.

## 1. Introduction

Ground penetrating radar (GPR) is a rapidly developing non-destructive detection technology in recent years, which has been widely used for underground road detection in urban areas. Compared with other underground road detection methods, it has the advantages of high detection efficiency, high accuracy, low cost, and simple on-site operation [1,2,3]. However, there are still some shortcomings in the practical application of GPR in road disease detection. High-frequency GPR signals have high detection accuracy but shallow detection depth, while low-frequency signals have deep detection depth but low detection accuracy. Processing and interpreting single-frequency radar data makes it difficult to achieve effective unity of detection depth and accuracy. Insufficient antenna frequency bandwidth results in insufficient resolution due to ringing interference, leading to misjudgment of disease targets. At present, the use of multi-frequency and multi-channel GPR systems for road detection mainly focuses on data superposition and comparison of spectra of different main frequency antennas. The resolution of the detection data has not been improved [4,5]. However, using GPR systems with different center frequencies for data fusion can effectively improve the performance of GPR underground detection [6,7].

GPR multi-frequency antenna data fusion is the integration of information from different main frequency antennas, utilizing their complementarity to obtain a comprehensive and detailed description of the same target or detection area in order to draw more accurate and reliable conclusions. Data fusion can synthesize new data that meets certain needs based on certain fusion algorithms, thereby obtaining further analysis and understanding of the detection area, as well as target detection, recognition, or tracking. Data fusion can enhance useful information in data, increase the reliability of data processing, obtain more accurate results, and make the system more practical [8].

Over the past decade, the data fusion algorithms proposed for application in the field of GPR mainly operated on the spatial domain methods of pixel points and the multi-scale and multi-resolution transformation domain methods. There has not yet been a model-based fusion method that accurately extracts data features using specific mathematical models. For example, Xiao et al. fused multi-frequency GPR data collected on the Qinghai–Tibet Railway using transform domain methods such as forward and inverse S-transform and deterministic deconvolution extrapolation (EDD) [9,10]; Feng et al. adopted the weight parameter of information entropy and the fusion criterion of weighted average for real-time spatial domain fusion of GPR data [11]; Xu et al. used the fusion method of information entropy as a weight and Fourier transform for GPR data fusion in the transform domain [12]. Later, Xu et al. conducted comparative fusion experiments on multi-frequency GPR data using four transform domain methods, Fourier transform, wavelet transform, S-transform, and principal component transform, and the results showed that wavelet transform fusion has more advantages [13]; Bi et al. studied three multi-frequency GPR data fusion algorithms: spatial domain weighted fusion/unweighted fusion (weighted average method) and transform domain weighted fusion (Fourier method) [14]. Based on the two-dimensional wavelet transform domain method, Lu et al. utilized a dynamic fusion weight scheme derived from edge detection algorithms to achieve better multi-frequency GPR data fusion images [15]; Xue et al. conducted fusion research on multi-polarization data of fully polarized GPR using principal component analysis, Laplace pyramid, and multi-scale wavelet transform methods [16]; Zhao et al. used genetic algorithms to fuse adaptive weights for multi-frequency GPR data and conducted fusion experiments using the weighted average spatial domain method [17]. Zhao et al. also studied the multi-frequency GPR data spatial domain fusion algorithm based on time-varying weighting strategy [18]; Shen et al. proposed a spatial domain fusion method for multi-frequency GPR data based on joint weighted deconvolution [19]; Liu et al. achieved effective transform domain fusion of multi-frequency GPR data using sliding window and wavelet transform weighted fusion method [20].

The spatial domain method that directly operates on pixel points is only a simple superposition operation, and the fusion rules will reduce the signal-to-noise ratio and contrast of the fusion signal. Traditional multi-scale transform domain methods use predefined fixed functions to extract features, such as spatial frequency and gradient energy, which lack the generalization of features [21,22]. In this article, in order to achieve feature adaptation, a mathematical model fusion method based on adaptive data feature extraction is proposed. We propose a multi-frequency GPR data fusion method based on convolutional sparse representation(CSR) to improve the imaging quality of GPR and the interpretation of underground detection. This article systematically adapts and applies the classic CSR multi-scale fusion framework to GPR data interpretation enhancement tasks for the first time by introducing customized fusion rules based on GPR signal characteristics, such as detail fusion based on structural consistency and base layer fusion based on information entropy. This solves the problem of insufficient physical targeting of general image fusion methods when directly processing GPR data. Firstly, the multi-frequency GPR data is spatially registered, and then the registered multi-frequency GPR data is CSR-fused using convolutional dictionary filters learned from simulated high-definition multi-frequency GPR data. Finally, the fused GPR data is reconstructed. Information entropy, average gradient, mutual information, and visual information fidelity were used as quantitative evaluation parameters to analyze the fusion results. The method was validated using simulated and real data, and the results were compared with those of traditional fusion methods based on weighted average (WA), principal component analysis (PCA), or two-dimensional wavelets (2D-WTs). This method can more effectively integrate the advantages of high- and low-frequency data, improve the imaging clarity and resolution of GPR, and greatly improve the imaging ability of GPR.

## 2. Technical Route

**Figure 1:**
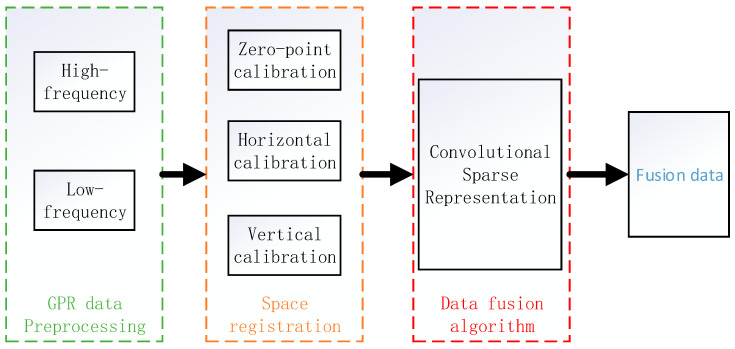
GPR data fusion process.

The data fusion methods of multi-frequency GPR can be divided into three types: data fusion obtained by multiple frequency antennas at different times, data fusion obtained by multiple frequency antennas at the same time, and data fusion obtained by a single-frequency antenna at different times or under different environmental conditions [12,13]. Through data fusion, useful information in the data can be strengthened, the reliability of data processing can be increased, and more accurate results can be obtained, which can further analyze, understand, and detect, identify, or track targets in the detection area. Its main fusion processes include data preprocessing, spatial registration, and data fusion. As shown in Figure 1.

The spatial consistency of the data to be fused is the guarantee for obtaining accurate output. Therefore, if the sampling rate, time window, or data frame interval of GPR data are different, it is necessary to perform operations on the data to be fused, and the main process of this operation is data preprocessing and spatial registration.

Even for the same target, the noise interference and time delay between multiple data sources in multi-frequency GPR data are different. In order to reduce the impact of noise, GPR data should undergo denoising preprocessing to improve signal-to-noise ratio. Denoising can be achieved through various methods, such as one-dimensional and two-dimensional filtering. To improve signal reflection intensity, gain is used for preprocessing to enhance clarity.

### 2.1. Space Registration

**Figure 2:**
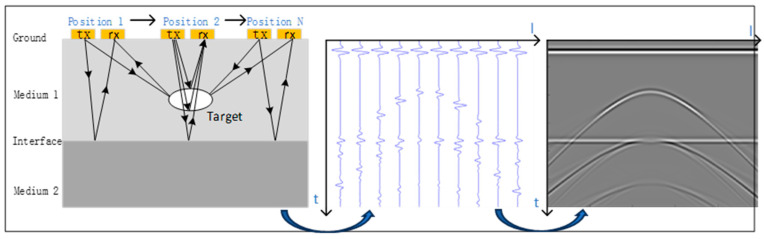
Schematic diagram of GPR waveform recording.

GPR graphics are often recorded in the form of pulse reflection waves, and the collected waveform is counted as one GPR data channel. The positive peak values in the waveform are marked in black and white, and the transition interval between the two is represented by a gradient gray level from black to white. In this way, the same phase axis, equal gray and equal-gray lines can vividly represent the underground reflection surface [23,24,25]. As shown in Figure 2, it is a schematic diagram of GPR waveform recording. Based on a simple geological model, a waveform record is drawn in the figure, and the reflection phase axis of the arc in the figure is the reflection surface of the target body.

After data preprocessing, it is necessary to correct the time delay between multiple frequency GPR data. In our study, the offset between the transmitting antenna and the receiving antenna was kept small enough to be negligible. Then, the above correction is achieved by aligning the data points to eliminate their time differences in the process we call zero-point calibration. Multi-frequency data spatial registration is a prerequisite for data fusion, and the accuracy of spatial registration directly determines the effectiveness of data fusion [26]. For GPR data, data space registration refers to establishing spatial correspondence between radar images of different frequencies in the same spatial coordinate system, including two aspects: horizontal calibration and vertical calibration. The purpose of horizontal calibration is not only to achieve consistent scanning of trace numbers for radar data of different frequencies, but also to ensure that data with the same trace number comes from the same horizontal position on the measurement line as much as possible. Vertical calibration needs to achieve the same sampling rate for radar data of different frequencies, and the number of sampling points for single channel data needs to be consistent after processing.

#### 2.1.1. Zero-Point Registration

Assuming there are K GPR data with different center frequencies, represented as $D_{k},k\in \left\{1,\;2,\ldots ,K\right\}$, whose data is represented as $D_{k}(i_{k},j_{k})$, where $i_{k}=1,\;2,\ldots ,M_{k}$, and the number of GPR data frames collected by $M_{k}$, $j_{k}$ = $1,\;2,\ldots ,N_{k},$ and $N_{k}$ are the number of sampling points for each data frame, the time window for each data frame is $t_{k}$, and the interval between each data frame is $∆l_{k}$.

Zero-point setting generally takes the time $t_{0}^{l}$ of the minimum value of the first arrival of each reflected wave in the response of radar data at different frequencies as the time zero point, where l is the position of the collected data frame. Zero-point registration refers to aligning the data points collected by the GPR antenna with a center frequency of f at the radar wave two-way time t to the time zero point $t_{0}^{l}$. The detailed calculation equation is as follows:

(1) $$\dot{D_{k}}\left(l,t-t_{0}^{l}\right)=D_{k}\left(l,t\right)$$

where $l=i_{k}∆l_{k},t=j_{k}\frac{t_{k}}{N_{k}}$.

#### 2.1.2. Interpolation Method

Several interpolation algorithms can be used for data calibration, such as nearest neighbor, bilinear interpolation, and cubic spline and Sinc interpolation [27]. Among them, interpolation based on Sinc shows superior performance compared to other methods and is commonly used for processing GPR data [28].

Let f(i),i=1,2…,I and $g\left(j\right),j=1,2,\ldots ,J$ represent the signals before and after interpolation, respectively. The normalized sinc function is given by the following equation

(2) $$sinc\left(t\right)=\frac{\sin\left(\pi t\right)}{\pi t}$$

where $sinc\left(0\right)=1$, the corresponding a fold interpolation is defined as

(3) $$g\left(m\right)=\sum_{i=1}^{I}f\left(i\right)sinc\left(\frac{j-ia}{a}\right),\;J=aI$$

when j is a multiple of i, $g\left(j\right)$ = $f\left(i\right)$.

#### 2.1.3. Horizontal Calibration

During the data acquisition process of GPR, the variation in acquisition speed can cause differences in track spacing, and non-uniform track spacing can cause horizontal deformation of the data. Therefore, it is necessary to register a uniform track spacing in the horizontal direction. Based on the maximum number of scanning traces in radar data of different frequencies, horizontal registration is achieved through interpolation, so that the number of radar profiles at different frequencies on the same measuring line is consistent. gcd( , ,) represents finding the maximum common divisor. So the multiples of the number of data channels inserted by the k-th frequency GPR are

(4) $$a_{k}^{s}=\frac{∆l_{k}}{\gcd\left(∆l_{1},\ldots ,∆l_{k}\right)}$$

So the number of GPR data channels for the k-th frequency after interpolation is $M_{k}^{*}=a_{k}^{s}M_{k}$, then the GPR data for the k-th frequency after horizontal interpolation is

(5) $$\dot{D_{k}^{*}}\left(i_{k}^{*},j_{k}\right)=\sum_{i_{k}=1}^{M_{k}}\dot{D_{k}}\left(i_{k},j_{k}\right)sinc\left(\frac{i_{k}^{*}-i_{k}a_{k}^{s}}{a_{k}^{s}}\right)$$

when $i_{k}^{*}$ is a multiple of $i_{k}$, $\dot{D_{k}^{*}}\left(i_{k}^{*},j_{k}\right)=\dot{D_{k}}(i_{k},j_{k})$, where $i_{k}^{*}=1,2,\ldots ,M_{k}^{*}$.

#### 2.1.4. Vertical Calibration

Due to the different measurement time windows of antennas with different frequencies, the sampling rate varies. Vertical registration requires interpolation of different frequency data to achieve the same sampling rate for different frequency radar data, and the number of sampling points for single channel data needs to be consistent after processing. $a_{k}^{c}$ is the multiple of the sampling point inserted for the k-th frequency GPR.

(6) $$a_{k}^{c}=\frac{t_{k}}{\gcd\left(t_{1},{\ldots ,t}_{k}\right)}$$

So the number of sampling points for the k-th frequency of the GPR after interpolation is $n_{k}^{*}=a_{k}^{c}n_{k}$, then the GPR data for the k-th frequency after horizontal interpolation is

(7) $$\dot{D_{k}^{**}}\left(i_{k}^{*},j_{k}^{*}\right)=\sum_{j_{k}=1}^{n_{k}}\dot{D_{k}^{*}}\left(i_{k}^{*},j_{k}\right)sinc(\frac{j_{k}^{*}-j_{k}a_{k}^{c}}{a_{k}^{c}})$$

when $j_{k}^{*}$ is a multiple of $j_{k}$, $\dot{D_{k}^{**}}\left(i_{k}^{*},j_{k}^{*}\right)=\dot{D_{k}^{*}}(i_{k}^{*},j_{k})$, where $j_{k}^{*}=1,2,\ldots ,n_{k}^{*}$.

### 2.2. Data Fusion

**Figure 3:**
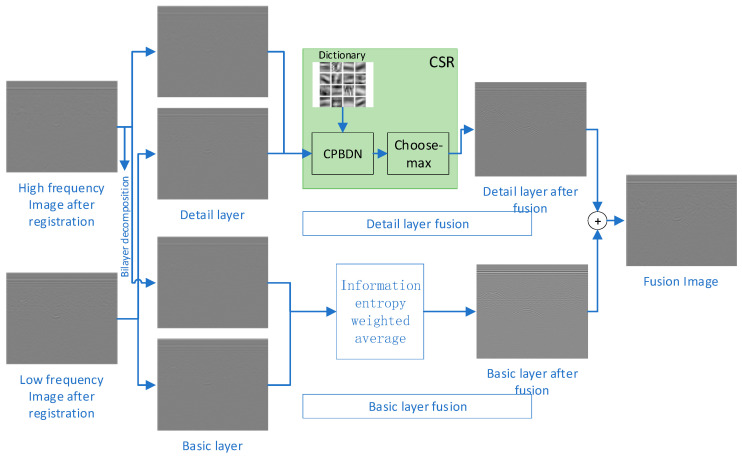
CSR multi-frequency GPR data fusion framework.

Figure 3 shows our method framework using grayscale images of multi-frequency GPR as an example. The framework mainly includes dual scale decomposition, detail layer fusion based on CSR, base layer fusion, and fusion reconstruction.

#### 2.2.1. Convolutional Sparse Representation

CSR can be seen as an alternative representation of sparse representation (SR) using convolution form, with the aim of achieving a sparse representation of the entire data [29]. Given the input signal data s and a set of filters of the same size $d_{m},m=1,2,\ldots ,M$, this set of filters is defined as a convolutional dictionary filter. Among them, * represents the convolution operator, λ is the sparse regularization term, $\|\;{\|}_{2}$ is the L-2 norm, and $\|\;{\|}_{1}$ is the L-1 norm. The basic idea of CSR is convolutional basis pursuit denoising (CPBDN) [30], which models the original signal s as the sum of a set of convolutions between the sparse coefficient map $x_{m}$ and the dictionary filter $d_{m}$:

(8) $$\underset{\left\{x_{m}\right\}}{\mathrm{argmin}}\frac{1}{2}{\left\|\sum_{m}d_{m}*x_{m}-s\right\|}_{2}^{2}+\lambda \sum_{m}{\left\|x_{m}\right\|}_{1}$$

Brendt Wohlberg’s research suggests that the alternating direction method of multipliers (ADMM) is the best solution to address the aforementioned optimization problems [31].

#### 2.2.2. Convolutional Dictionary Filters Learning

The convolutional dictionary filters need to be learned from training samples [32,33,34], $s_{k},k\in \{1,2,\ldots ,K\}$ is the set of training sample data, $d_{m},\;m\in \left\{1,2,\ldots ,M\right\}$ is the convolutional dictionary filter to be learned, and $x_{k,m}$ is a sparse coefficient map. And the convolutional dictionary filter learning model can be written as the following optimization objective function:

(9) $$\underset{\left\{d_{m}\right\}\left\{x_{k,m}\right\}}{\mathrm{argmin}}\frac{1}{2}\sum_{k}{\left\|\sum_{m}d_{m}*x_{k,m}-s_{k}\right\|}_{2}^{2}+\lambda \sum_{k}\sum_{m}{\left\|x_{k,m}\right\|}_{1}$$

The constraint of ${\left\|d_{m}\right\|}_{2}=1$ on the specification of convolutional dictionary filters is to avoid scale ambiguity between convolutional dictionary filters and sparse coefficient mappings. Due to the inevitable presence of noise in real GPR data, which can have an impact on the training results of convolutional dictionaries, in order to reduce the impact of these noises, we constructed a uniform medium road model, added holes or looseness of different sizes at different depths, and used GPR antennas of different frequencies to obtain noise free simulated data. Use simulation software for GprMax 3.0. GprMax is an open source software that simulates electromagnetic wave propagation. It solves Maxwell’s equations in 3D using the Finite-Difference Time-Domain (FDTD) method. GprMax was designed for modeling GPR.

**Figure 4:**
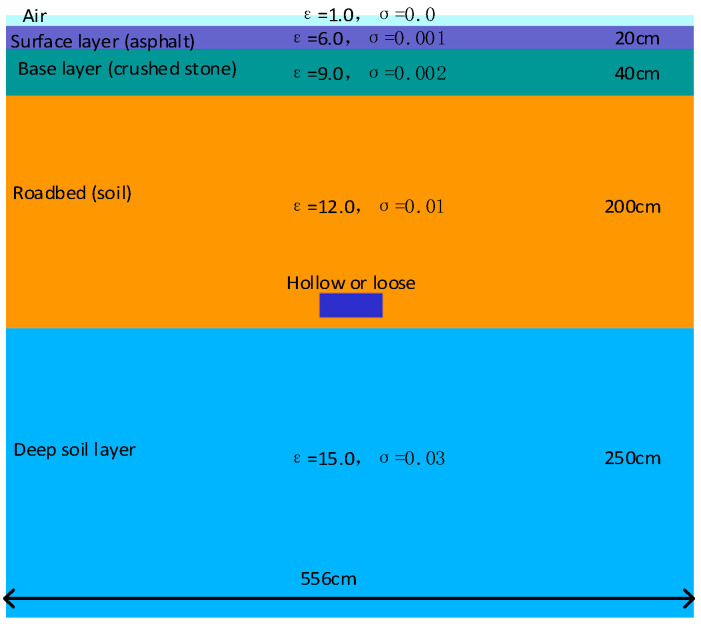
Slicing of uniform medium road model.

The simulation of urban roads should be as close as possible to the real urban road structure. This study is based on the “Technical Standard for Highway Engineering in Suburban and Rural Town Areas” JTG 2112-2021, published in China [35]. As shown in Figure 4, urban roads are divided into three parts from top to bottom: surface layer, base layer, and soil foundation. In order to improve the clarity of dictionary training images, the thickness of each layer of media is uniform to reduce noise generation. The depth of the model established in the experiment is 5.1 m. ε represents the relative dielectric constant, and σ is the conductivity.

**Figure 5:**
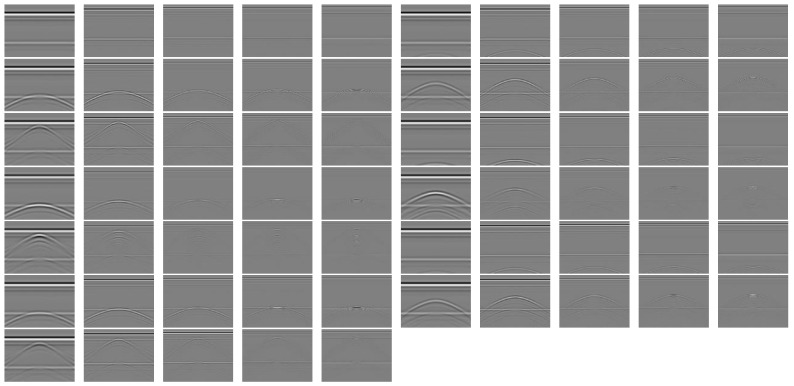
Simulated road GPR grayscale atlas.

By simulating antennas with center frequencies of 100 MHz, 200 MHz, 300 MHz, 400 MHz, and 500 MHz, 65 512 × 512 GPR data were obtained at different depths (3.5 m, 2.5 m, 1.5 m, and 0.5 m) with hollow or two sets of looseness, or with neither hollow nor looseness. The obtained grayscale image is shown in Figure 5. From left to right, every 5 images correspond to 5 types of antennas. The first 5 images have no hollow or looseness, the next 20 images have hollow of different depths, the next 20 images have the first looseness of different depths, and the next 20 images have the second looseness of different depths. Train CSR convolutional dictionary filters using high-definition multi-frequency GPR data.

**Figure 6:**
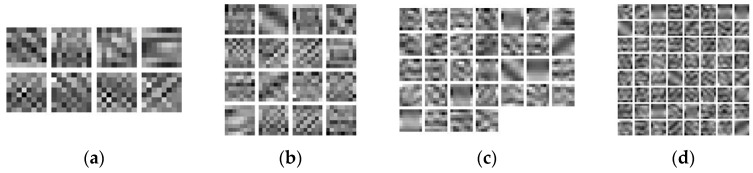
Different lengths of convolutional dictionary filters (8 × 8): (**a**) Length 8, (**b**) Length 16, (**c**) Length 32, (**d**) Length 64.

**Figure 7:**
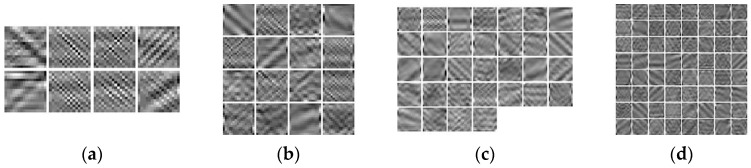
Different lengths of convolutional dictionary filters (16 × 16): (**a**) Length 8, (**b**) Length 16, (**c**) Length 32, (**d**) Length 64.

Based on the simulated high-definition data in Figure 5, the size of the convolutional dictionary filters was fixed to 8 × 8, and the length of the convolutional dictionary was 8, 16, 32, and 64. The convolutional dictionary was trained as shown in Figure 6. The size of the convolutional dictionary filters was fixed at 16 × 16, with lengths of 8, 16, 32, and 64. The convolutional dictionary filters was trained as shown in Figure 7.

#### 2.2.3. Data Fusion Process

Step 1: Double-layer decomposition

Firstly, decompose the GPR data $\dot{D_{k}^{**}}$ after registering each frequency into the basic layer $D_{k}^{b}$ and the detail layer $D_{k}^{d}$. Calculate the F norm for $\|\;{\|}_{F}$, with $g_{x}=\left[-1,\;1\right]\;$ and $\;\;\;g_{y}={\left[-1,\;1\right]}^{T}$ as the horizontal and vertical gradient operators, and η as the regularization parameter. Obtain the detail layer by solving the following optimization problem:

(10) $$\underset{\left\{D_{k}^{l}\right\}}{\mathrm{argmin}}{\left\|\dot{D_{k}^{**}}-D_{k}^{d}\right\|}_{F}^{2}+\eta \left({\left\|g_{x}*D_{k}^{d}\right\|}_{F}^{2}+{\left\|g_{y}*D_{k}^{d}\right\|}_{F}^{2}\right)$$

This is a Tikhonov regularization problem that can be effectively solved through two-dimensional fast Fourier transform. Obtain the base layer by subtraction:

(11) $$D_{k}^{b}=\dot{D_{k}^{**}}-D_{k}^{d}$$

Step 2: Fusion of detail layers

For each detail layer $D_{k}^{d}$, its sparse coefficient maps to $C_{k,m},m\in \{1,...,M\}$. The alternating direction method (ADMM) of the multiplier is used to solve the CSR model, resulting in:

(12) $$\underset{\left\{C_{k,m}\;\right\}}{\mathrm{argmin}}\frac{1}{2}{\left\|\sum_{m=1}^{M}d_{m}*C_{k,m}-D_{k}^{d}\right\|}_{2}^{2}+\lambda \sum_{m=1}^{M}{\left\|C_{k,m}\right\|}_{1}$$

Let $C_{k,1:M}(x,y)$ represent the data value of $C_{k,m}$ at position (x,y) in the spatial domain. Obviously, $C_{k,1:M}(x,y)$ is a dimensional vector. According to the fusion method based on SR [26], the L1 norm of $C_{k,1:M}(x,y)$ is used as the activity level metric for the source image. Therefore, the activity level map $A_{k}(x,y)$:

(13) $$A_{k}\left(x,y\right)={\left\|C_{k,1:M}\left(x,y\right)\right\|}_{1}$$

In order to make the method insensitive to mismatches, a window-based averaging strategy is executed on $A_{k}(x,y)$ to obtain the final activity level map:

(14) $$\bar{A_{k}}\left(x,y\right)=\frac{\sum_{p=-r}^{r}\sum_{q=-r}^{r}A_{k}\left(x+p,y+q\right)}{{\left(2r+1\right)}^{2}}$$

Finally, reconstruct the fusion results of the detail layer:

(15) $$D_{F}^{d}=\sum_{m=1}^{M}d_{m}*C_{f,m}$$

Step 3: Basic layer fusion

However, this selection strategy may lead to visual inconsistency in images of GPR data at different frequencies, as the grayscale values at the same location may differ significantly. Therefore, the information entropy weight strategy is applied to the fusion of the basic layer of multi-frequency GPR data. If the information entropy of the k-th GPR after registration is $E_{k}$, then the fusion result of the base layer is:

(16) $$D_{F}^{b}\left(x,y\right)=\frac{\sum_{k=1}^{K}{E_{k}D}_{k}^{l}\left(x,y\right)}{\sum_{k=1}^{K}E_{k}}$$

Step 4: Reconstruction

Take the fused basic layer $D_{F}^{b}$ and detail layer $D_{F}^{d}$, and reconstruct the fused data $D_{F}$.

(17) $$D_{F}={D_{F}^{b}+D}_{F}^{d}$$

## 3. Experimental Analysis

### 3.1. Evaluation Criteria

#### 3.1.1. Information Entropy

The information entropy (IE) of the fused image is an important parameter for measuring the increase in information content of the fused image [12]. The larger the information entropy value, the richer the information contained in the fused image. So, the effect of image fusion can be evaluated by comparing the changes in image information entropy before and after fusion. p(i) is the probability of the grayscale value i appearing in the image, and L is the grayscale level of the image. The calculation formula is

(18) $$IE=-\sum_{i=0}^{L-1}p(i)\log_{2}p(i)$$

#### 3.1.2. Average Gradient

The average gradient (AG) refers to the rate of change in grayscale near the boundaries or shadows of an image, and the magnitude of this rate of change can be used to represent the clarity of the image [13]. It reflects the rate of contrast change in small details of an image and is an important indicator of image clarity and the ability to express image details. The larger the average gradient, the clearer the image. $F\left(i,j\right)$ is the grayscale value of the i-th row and j-th column of the image; M and N represent the total number of rows and columns, respectively. The calculation formula is

(19) $$AG=\frac{1}{\left(M-1\right)\left(N-1\right)}\times \sum_{i=1}^{M-1}\sum_{j=1}^{N-1}\sqrt{\frac{{\left(F\left(i,j\right)-F\left(i+1,j\right)\right)}^{2}+{\left(F\left(i,j\right)-F\left(i,j+1\right)\right)}^{2}}{2}}$$

#### 3.1.3. Mutual Information

Mutual Information (MI) is a useful measure of information in information theory, which refers to the correlation between two sets of events [36,37,38,39]. The greater the mutual information, the better the fusion quality, as the fused image retains more source image information. For the grayscale images of a and b, their information entropy is $E_{a}$ and $E_{b},$ respectively, and their joint information entropy is $E_{ab}$. p(i,j) is the probability of the grayscale value (i,j) appearing in the image, i is the grayscale value of image a, j is the grayscale value of image b, and L is the grayscale level of the image. Therefore, the formula for calculating mutual information is

(20) $$MI=E_{a}+E_{b}-E_{ab}$$

(21) $$E_{ab}=-\sum_{i=0}^{L_{a}-1}\sum_{j=0}^{L_{b}-1}p(i,j)\log_{2}p(i,j)$$

#### 3.1.4. Visual Information Fidelity

Visual information fidelity (VIF) is a new criterion based on natural scene statistics (NSS), image distortion, and human visual distortion modeling [31]. This indicator assumes that the image seen by the human eye is information filtered out through HVS (Hue, Saturation, Value), Hue (H), Saturation (S), and Brightness (V). HVS itself is a distortion channel, that is, the human visual distortion channel. Distorted images only pass through an additional image distortion channel before passing through HVS, so the knowledge of information theory can be used to compare the information extracted by the human eye with the information extracted from the original image, and obtain the final evaluation result. Specifically, the reference image is modeled through the HVS channel and then output from a random “natural” source processed by the brain. The information of the reference image is quantified as mutual information between the input and output of the HVS channel, which is the most ideal information that the brain can extract from the HVS output. Then, let the same reference image pass through the distortion channel and quantify the measurement. Combine these two pieces of information to form a visual information fidelity that is related to visual quality and relative image information. The range of VIF values is [0, 1], and the larger the VIF value, the better the quality of the fused image.

### 3.2. Simulation Experiments

**Figure 8:**
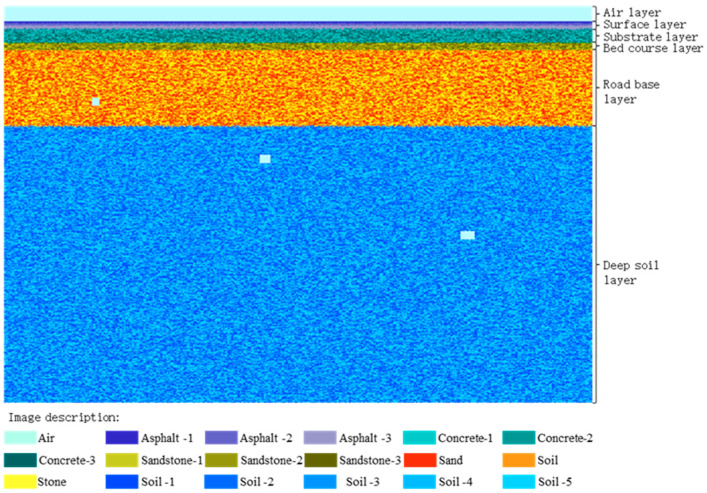
Vertical slicing of road models.

To verify the effectiveness of CSR in GPR data fusion, a simulation model was designed to collect GPR data at different frequencies. In order to approach the real road structure more closely, a more refined design is carried out for each layer of the road. As shown in Figure 8, below the air layer is a general road structure, including surface layer, base layer, cushion layer, roadbed layer, and deep soil layer. The experiment uses pixels of different sizes to simulate corresponding material particles. The random stacking of different particles forms different shapes, making the material shape and size more random. In simulated data, the non-uniformity of real environmental media and the randomness of electromagnetic wave propagation can also be well simulated. The model established in the experiment has a depth of 10.4 m and a length of 17.4 m. The relevant parameters of the dimensions and materials of other road models are shown in Table 1. Based on the parameters in the table, simulate the non-uniform medium in the real underground environment by randomly distributing the corresponding materials in each layer. The cavity is located at different depths and has different lengths. As the depth increases, the length of the cavity is 0.2 m, 0.3 m, and 0.4 m, respectively. The vertical slice description example of the road model and the material legend corresponding to each color are shown in Figure 8.

**Figure 9:**
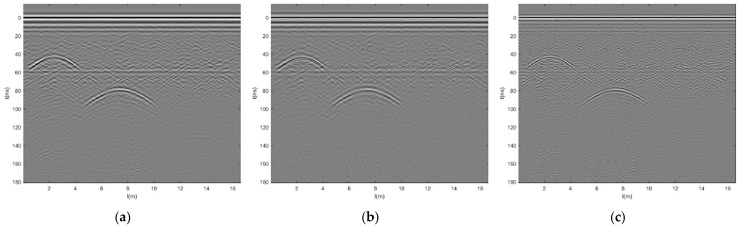
GPR grayscale image after registration. (**a**) 100 MHz, (**b**) 200 MHz, and (**c**) 400 MHz.

Using GPR antennas with frequencies of 100 MHz, 200 MHz, and 400 MHz, respectively, with time windows of 170 ns and data channel intervals of 0.02 m, after the same data processing process and spatial registration, the multi-frequency GPR data grayscale images are shown in Figure 9a–c. From the figure, it can be seen that the 100 MHz antenna has weaker resolution for surface and base layer layering, and clearer resolution for deep voids. The 200 MHz and 400 MHz antennas have clearer layering for the surface layer and base layer and are not clear for deep voids. The 400 MHz antenna cannot detect deeper layers. Verified that GPR has higher high-frequency resolution and deeper low-frequency detection depth.

According to the fusion process of CSR, different convolutional dictionary filters, η, λ, and r have an impact on the fusion effect of multi-frequency GPR. It is necessary to analyze these different parameters separately. We obtained the optimal parameters through simulating multi-frequency GPR experiments to prepare for achieving better results in later real experiments.

To explore the impact of convolutional dictionary filters on the fusion performance of multi-frequency GPR data, eight sets of learned convolutional dictionary filters were used to fuse the registered 100 MHz, 200 MHz, and 400 MHz simulated GPR data pairwise. The 100–200 MHz represents the fusion data of 100 MHz and 200 MHz GPR; 100–400 MHz represents the fusion data of 100 MHz and 400 MHz GPR; and 200–400 MHz represents the fusion data of 200 MHz and 400 MHz GPR. The fixed η is 5, the fixed λ is 0.001, and the fixed r is 3.

**Figure 10:**
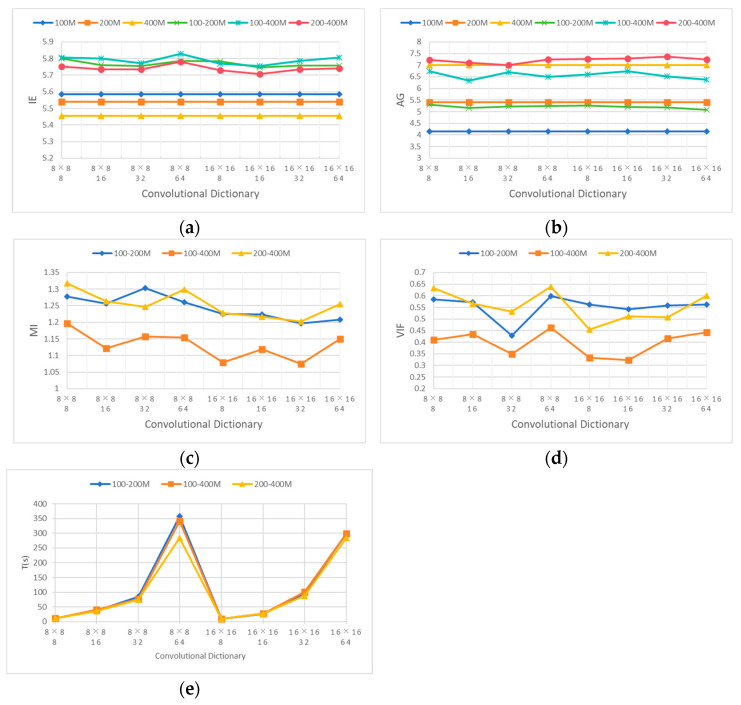
Different convolutional dictionary filters on the evaluation criteria curves of fused data. (**a**) IE, (**b**) AG, (**c**) MI, (**d**) VIF, and (**e**) time.

From the evaluation criteria curves of each fusion in Figure 10, the following results can be obtained. The IE curve in Figure 10a shows that under the same length, the fusion effect of the 8 × 8 convolutional dictionary filter is better than that of the 16 × 16 convolutional dictionary filter, with a length of 64 reaching the best. Considering the fusion time, choosing the convolutional dictionary filter with a length of 8 is better. The AG in Figure 10b is less affected by different convolutional dictionary filters. As the length of the convolutional dictionary filter increases, the fusion time also increases. Convolutional dictionaries with lengths of 8 and 16 were chosen. The MI curve in Figure 10c shows that under the same length, the fusion effect of the 8 × 8 convolutional dictionary filter is better than that of the 16 × 16 convolutional dictionary filter. The fusion effects of 100–400 MHz and 200–400 MHz are the best at length 8, and the best is achieved at length 32 from 100 MHz to 200 MHz. Considering time cost, the 8 × 8 8 convolutional dictionary filter is selected. From the VIF curve in Figure 10d, it can be seen that under the same length, the fusion effect of the 8 × 8 convolutional dictionary filter is better than that of the 16 × 16 convolutional dictionary filter. Considering the time curve in Figure 10e, 100–200 MHz and 200–400 MHz are better at length 8; we choose the 8 × 8 8 convolutional dictionary filter. So it is more appropriate to choose an 8 × 8 8 convolutional dictionary filter from these evaluation result curves. In the following figures, 200–400 M represents the frequency range of 200–400 MHz.

**Figure 11:**
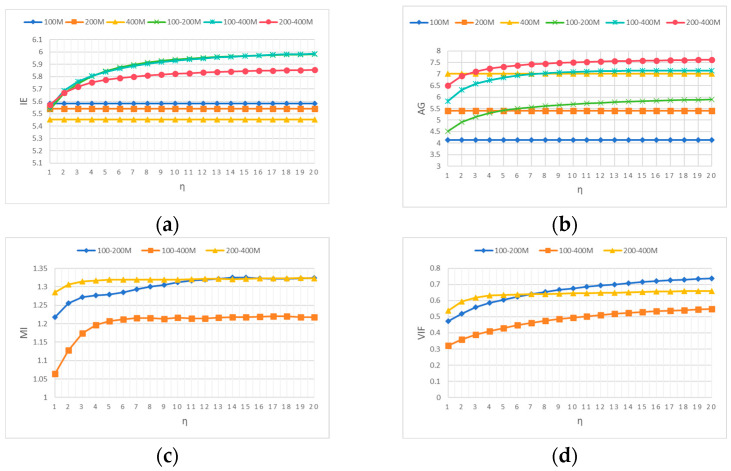
Different evaluation criteria curves for fused data with different η values. (**a**) IE, (**b**) AG, (**c**) MI, and (**d**) VIF.

To explore the influence of η on the fusion performance of multi-frequency GPR data, an 8 × 8 8 convolutional dictionary filter was selected, with a fixed λ of 0.001 and a fixed r of 3. From the curves of various evaluation results in Figure 11a–d, it can be seen that as the value of η increases, EI, AG, MI, and VIF also increase. In the AG curve, the fusion results of 100–400 MHz are better than the original image after the value of η exceeds 8. However, if the value of η is too large, the information contained in the detail layer is less. CSR is mainly the fusion of the detail layer, so η is selected as 8.

**Figure 12:**
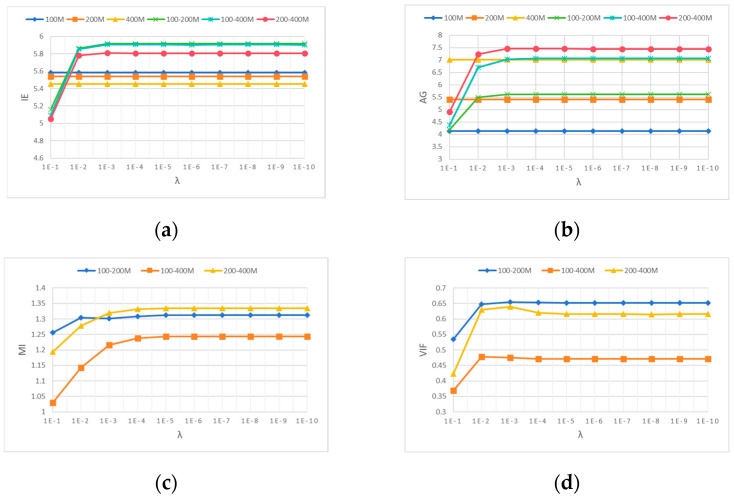
Curve of evaluation criteria for fused data with different λ values. (**a**) IE, (**b**) AG, (**c**) MI, and (**d**) VIF.

To explore the impact of λ on the fusion performance of multi-frequency GPR data, an 8 × 8 8 convolutional dictionary filter was selected, with a fixed value of 8 for η and 3 for r. From the curves of each evaluation result in Figure 12a–d, it can be seen that as λ decreases, EI, AG, MI, and VIF all increase. After λ is 0.001, the curves of each evaluation result tend to stabilize, so λ is selected as 0.001. 1E-3 in Figure 12 represents 1 × 10^−3^, while others are represented in this way.

**Figure 13:**
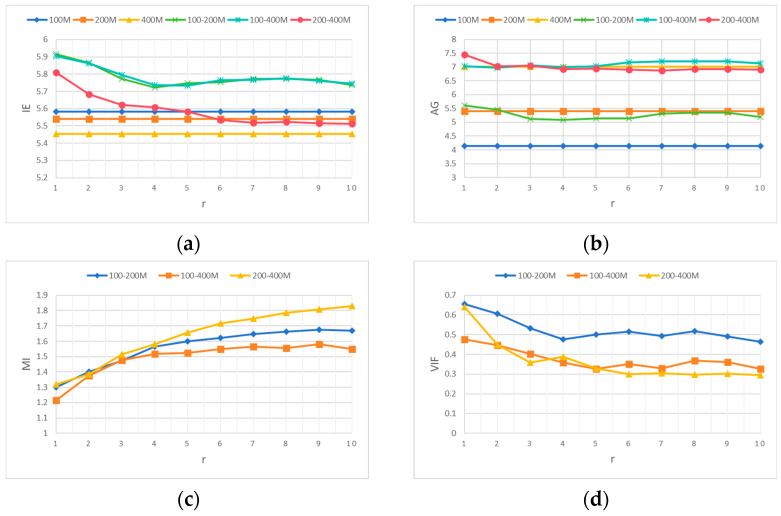
Curve of evaluation criteria for different r pairs of fused data. (**a**) IE, (**b**) AG, (**c**) MI, and (**d**) VIF.

To explore the impact of r on the fusion performance of multi-frequency GPR data, an 8 × 8 8 convolutional dictionary filter was selected, with a fixed η of 8 and a fixed λ of 0.001. From the evaluation result curves in Figure 13a–d, it can be seen that as r increases, EI, AG, and VIF all decrease, while MI increases, where r determines the window size. This method has strong robustness against larger mismatches but may also lose some small details. In multi-frequency GPR data fusion, it is more appropriate to use a smaller r due to the emphasis on small-scale details. Select r as 1 based on the EI and VIF curves.

**Figure 14:**
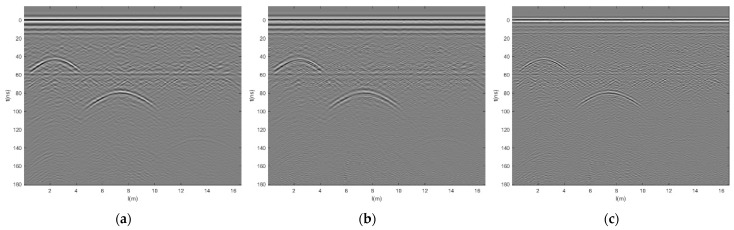
Simulated multi-frequency GPR fusion map. (**a**) 100 MHz and 200 MHz fusion map, (**b**) 100 MHz and 400 MHz fusion map, and (**c**) 200 MHz and 400 MHz fusion map.

Based on the analysis of the fusion results of multi-frequency GPR using various parameters, it can be concluded that selecting an 8 × 8 convolutional dictionary filter, with a value of η is 8, λ is 0.001, and r is 1, can obtain the best fusion evaluation standard result. Figure 14a–c shows the grayscale images of 100 MHz, 200 MHz, and 400 MHz GPR data paired with CSR fusion using the selected optimal parameters. In each fusion image, shallow voids are clearer, deep voids are also more prominent, and shallow layering is clearer, including the high resolution of high-frequency GPR and the deep detection depth of low-frequency GPR.

Specifically, WA fusion, PCA fusion, 2D-WT fusion, and CSR fusion were performed on simulated multi-frequency GPR data after data registration. Among the evaluation indicators listed in Table 2, CSR was the best compared to WA, PCA, and 2D-WT. The information entropy of the 100 MHz, 200 MHz, and 400 MHz raw data are 5.5839, 5.5392, and 5.4533, respectively, with an average gradient of 4.1415, 5.4048, and 7.0083. As listed in Table 2, the information entropy and average gradient of the fused GPR are higher than those of the original data, indicating that the fused data is better.

### 3.3. Real Experiments

#### 3.3.1. Data Collection 

**Figure 15:**
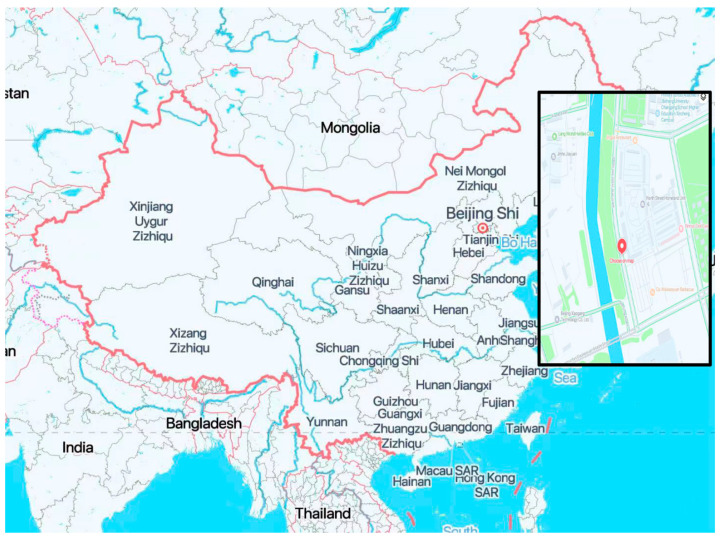
Road measurement location.

To evaluate the performance of the algorithm, we conducted detection experiments on the road using a multi-frequency and multi-channel array GPR system from China University of Mining and Technology (Beijing), with GPR antennas with center frequencies of 200 MHz and 400 MHz, respectively. The road measurement location is shown in Figure 15.

#### 3.3.2. Data Processing

**Figure 16:**
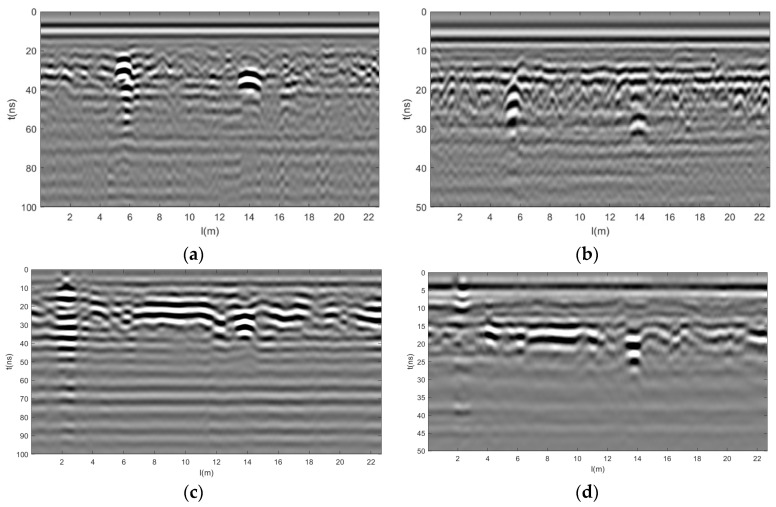
Gray scale image of GPR data for two road sections. (**a**) Section 1 200 MHz, (**b**) Section 1 400 MHz, (**c**) Section 2 200 MHz, and (**d**) Section 2 400 MHz.

The time windows were configured to be 100 ns and 50 ns, with 512 sampling points and a channel interval of 0.0227 m. After one-dimensional and two-dimensional filtering and gaining preprocessing of the raw data, the grayscale images of the 200 MHz and 400 MHz GPR data were obtained as shown in Figure 16. The 20–60 ns resolution at 200 MHz is very good, as shown in Figure 16a,c, while the 10–30 ns resolution at 400 MHz is even better, as shown in Figure 16b,d.

**Figure 17:**
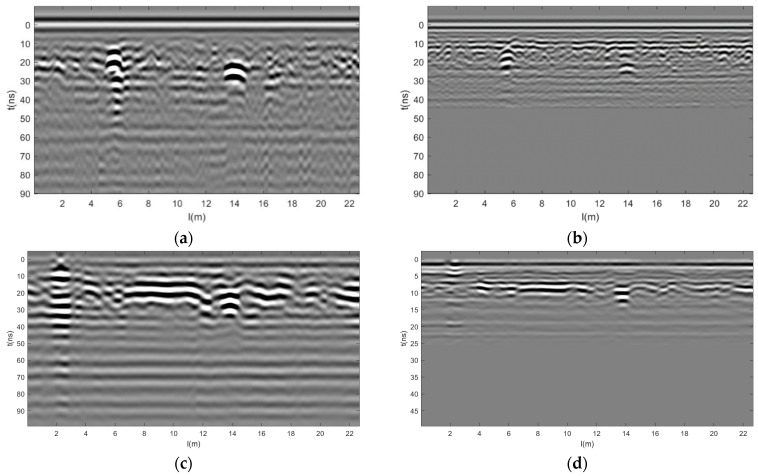
Gray scale image of GPR data after registration of two road sections. (**a**) Section 1 200 MHz, (**b**) Section 1 400 MHz, (**c**) Section 2 200 MHz, and (**d**) Section 2 400 MHz.

Consider GPR data at 200 MHz and 400 MHz, with 512 sampling points per trace and time windows of 100 ns and 50 ns, respectively. After zero-point registration, the GPR data of different frequencies are unified into the same coordinate system, and the 400 MHz data points are shifted downwards for later data fusion by filling the front zeros of the 400 MHz data. After spatial registration, to maintain the same sampling rate between 400 MHz data and 200 MHz data, each trace of 200 MHz GPR data has 1024 sampling points, and each trace of 400 MHz data has 512 sampling points. For later data fusion, 400 MHz increases the number of sampling points to 1024 by padding the data with zero at the end. After registration processing (Figure 17), the resolution of 0–60 ns at 200 MHz is very good, as shown in Figure 17a,c. And the resolution of 0–30 ns at 400 MHz is better, as shown in Figure 17b,d.

#### 3.3.3. Data Fusion Processing

**Figure 18:**
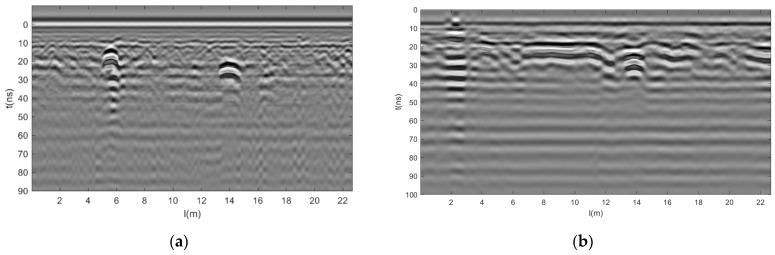
CSR fusion GPR data grayscale image. (**a**) Section 1 and (**b**) Section 2.

As shown in the gray scale diagram of GPR data after fusion in Figure 18, the shallow stratification and anomalies of GPR data are more obvious after CSR fusion, and the deep anomalies are more obvious after 30 ns. Compared with the grayscale images of 200 MHz and 400 MHz GPR data, the fused image resolution is improved, and the detailed information is more abundant.

#### 3.3.4. Evaluation of Fusion Results

From Table 3, it can be seen that compared with weighted average, principal component analysis, and two-dimensional wavelet, convolutional sparse fusion has higher information entropy, average gradient, visual information fidelity, clarity, resolution, and better data image quality. The mutual information CSR algorithm is not the highest, but it is only better than the weighted average algorithm, indicating that in real data, CSR fusion data is also better.

## 4. Conclusions

This study successfully constructed an adaptive multi-frequency GPR data fusion model based on CSR. Through the application of spatial registration, convolutional dictionary filtering learning, and customized fusion rules, this method has demonstrated superior performance in both simulated and measured data. The quantitative evaluation indicators (information entropy, average gradient, mutual information, visual information fidelity) show that, compared with traditional fusion methods such as weighted average, principal component analysis, and two-dimensional wavelet, the proposed method has significant advantages in preserving structural features, enhancing detail information, and improving physical consistency, effectively improving the interpretation quality and imaging effect of GPR data.

This study has the following limitations: Convolutional dictionary filtering learning relies on simulated high-frequency data, and the matching degree with actual scenes may affect the fusion generalization ability. The adaptability of the designed fusion rules to complex underground structures and noise interference still needs to be verified. The method has high computational complexity and faces efficiency challenges when processing large-scale data.

Future work can be carried out from four aspects: exploring a more robust dictionary learning mechanism that integrates prior knowledge of measured data; researching dynamic fusion rules that adapt to different geological conditions and noise levels; optimizing algorithm efficiency through parallel computing or lightweight design; and expanding to 3D GPR data fusion and multi-sensor joint interpretation applications.

## Figures and Tables

**Table 1:** Road model dimensions and material electrical performance parameters.

Layer Name	Thickness (m)	Material	$\mathrm{Relative}\;\mathrm{Dielectric}\;\mathrm{Constant}\;\varepsilon_{r}$	Conductivity σ (S/m)
Air layer	0.40	Air	1	0
Surface layer	0.06–0.08	Asphalt-1	3.1	0.004
	0.06–0.08	Asphalt-2	4.5	0.006
	0.06–0.08	Asphalt-3	5.9	0.008
Substrate layer	0.36–0.40	Concrete-1	7.2	0.008
		Concrete-2	7.8	0.0082
		Concrete-3	8.3	0.009
Bed course layer	0.18–0.22	Sandstone-1	8.5	0.008
		Sandstone-2	9.3	0.0095
		Sandstone-3	9.2	0.0092
Road base layer	1.96–2.04	Sand	10.2	0.0204
		Soil	11.4	0.0228
		Stone	12.2	0.0244
Deep soil layer	7.20–7.24	Soil-1	13.8	0.04
		Soil-2	14.2	0.0412
		Soil-3	14.0	0.0406
		Soil-4	14.8	0.0429
		Soil-5	15.5	0.0465
Cavity	0.20	Air	1	0

**Table 2:** Evaluation index values for various fusion algorithms of simulated multi-frequency GPR data.

High and Low Frequencies	Algorithm	IE	AG	MI	VIF
100 MHz and 200 MHz	WA	5.2574	3.6793	1.1621	0.3977
	PCA	4.9175	3.3646	0.7252	0.2208
	2D-WT	5.2584	3.6836	1.1625	0.3979
	CSR	**5.9173**	**5.6172**	**1.3003**	**0.6546**
100 MHz and 400 MHz	WA	5.2353	4.1882	1.0150	0.3186
	PCA	4.9397	3.4162	0.7155	0.2424
	2D-WT	5.2423	4.2226	1.0141	0.3198
	CSR	**5.9056**	**7.0379**	**1.2149**	**0.4757**
200 MHz and 400 MHz	WA	5.2933	5.2893	1.2630	0.4490
	PCA	4.8477	3.9765	0.7615	0.3029
	2D-WT	5.2970	5.3029	1.2551	0.4490
	CSR	**5.8119**	**7.4593**	**1.3194**	**0.6389**

**Table 3:** Evaluation index values of various fusion algorithms for real data.

Road Section	Algorithm	IE	AG	MI	VIF
1	WA	6.3504	1.7274	**1.6735**	0.3932
	PCA	6.2238	1.5318	0.8480	0.2818
	2D-WT	6.3521	1.7652	1.6411	0.3933
	CSR	**6.4771**	**2.0829**	1.6521	**0.4847**
2	WA	6.8045	1.7070	3.7465	0.4261
	PCA	6.6482	1.5218	2.2743	0.3045
	2D-WT	6.8057	1.7394	3.6456	0.4261
	CSR	**6.8589**	**1.8867**	**3.8257**	**0.4817**

## Data Availability

The original contributions presented in this study are included in the article. Further inquiries can be directed to the corresponding author.
